# Facelift Randomized Controlled Trials Compliance With CONSORT Checklist: A Systematic Review

**DOI:** 10.1093/asjof/ojaf153

**Published:** 2025-11-27

**Authors:** Yousif F Yousif, Wameth Alaa Jamel, Rocio Perez H, Omar Farooq, Elif Betül Balcı, Valeria de la Torre, Maryam I Darr, Kian Daneshi, Ankur Khajuria

## Abstract

**Background:**

Randomised controlled trials (RCTs) in facelift surgery remain few and variably reported; transparent, reproducible methods are essential to interpret efficacy and safety.

**Objectives:**

To assess the methodological quality and reporting standards of facelift RCTs using the Consolidated Standards of Reporting Trials (CONSORT) and CONSORT-NPT guidelines, identifying patterns of adherence and areas for improvement.

**Methods:**

We conducted a PRISMA-guided systematic review of RCTs evaluating facelift techniques or perioperative strategies. RCTs focusing on facelift techniques were included based on study design and relevance. Adherence to the CONSORT 2010 or CONSORT-NPT 2017 checklist was retrospectively assessed for each included study. Risk of bias was assessed using the Cochrane RoB 2.0 tool, and evidence quality was appraised via GRADE.

**Results:**

Ten RCTs (n = 457; mean sample 46) met inclusion. Mean CONSORT adherence was 56%, with high for intervention description and statistical analysis (both 100%) but poor for tailored interventions (10%), trial registration (20%), and trial protocol (30%). Adherence showed weak correlations with journal impact factor (*R*² = 0.0024) and author count (*R*² = 0.171). Only 3 trials were low risk of bias; GRADE certainty was largely low-moderate, limited by imprecision and suspected publication bias.

**Conclusions:**

Facelift RCTs show variable, often suboptimal reporting, leaving the evidence base thin despite rising demand. Strengthening trial quality requires field-wide pre-registration and protocol publication, validated outcome measures, and consistent CONSORT enforcement; a standardized minimum dataset and registry-based reporting would further bolster evidence for facial rejuvenation surgery.

**Level of Evidence: 5 (Therapeutic):**

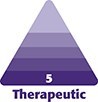

Facelift surgery, also known as rhytidectomy, is one of the most commonly performed cosmetic procedures, aimed at improving facial aesthetics and restoring youthful appearance.^1^ According to the American Academy of Facial Plastic and Reconstructive Surgery (AAFPRS), in 2023, members performed an average of 48 facelifts or partial facelifts per surgeon, representing a 60% increase since 2017.^2^ This is further supported by the 2023 American Society of Plastic Surgeons (ASPS) Procedural Statistics Report, where the number of facelift procedures performed in 2023 increased by 8% compared to 2022, indicating a notable year-over-year growth in demand for surgical facial rejuvenation.^3^ Despite its widespread use, high-quality evidence guiding best practices remains limited. Case series and expert opinion have traditionally dominated the field, making it difficult to draw strong, generalisable conclusions about surgical efficacy and safety.^4^

Randomized controlled trials, considered the gold standard for establishing causality in clinical research, are increasingly being applied in plastic and reconstructive surgery.^4,5^ Their role is particularly important in a field like facial aesthetic surgery, where outcomes are nuanced, subjective, and influenced by multiple variables.^6^ However, RCTs in surgery present unique challenges, including variability in surgical skill, complexity of procedures, and ethical considerations around blinding and control groups.^7,8^ To improve the transparency and quality of RCT reporting, the Consolidated Standards of Reporting Trials (CONSORT) statement was developed in 1996, with a 2008 extension for non-pharmacological treatments (CONSORT-NPT) addressing surgical interventions.^9^ Despite these guidelines, studies continue to show suboptimal adherence, especially in surgical specialties like plastic surgery.^10^

Given the increasing use of RCTs in facelift research and the methodological challenges they entail, evaluating compliance with the CONSORT guidelines is crucial.^11^ As a diverse speciality, it presents unique challenges for surgeons. Policymakers, patients and insurers seek evidence-based answers, and systematic reviews and meta-analyses provide the highest quality evidence. They allow summarizing and critically appraising the available evidence to inform practice guidelines, identify knowledge gaps, define surgical quality metrics, and guide resource allocation, which is essential, especially in the specific area of facelift articles with high-level evidence still lacking^11,12^.

As no systematic review has specifically evaluated CONSORT compliance in facelift RCTs, this review aims to assess the quality of RCT reporting across all eligible studies to provide a critical appraisal of methodological transparency in the field, by identifying common deficiencies, analyzing adherence patterns, and highlighting areas where improved reporting may enhance the utility of RCTs in informing clinical practice.^8^ Addressing these gaps can help improve methodological rigor, enhance reproducibility, and ultimately strengthen the quality and reliability of the research base that informs surgical practice.^11^

Importantly, the outcomes that matter most in facial aesthetic surgery are often subjective by nature. Our intent is not to privilege “objective” endpoints over expert observation or patient-reported experience, but to assess whether trials, regardless of endpoint type, report their methods with sufficient clarity to enable appraisal, replication, and synthesis. In this sense, CONSORT provides a flexible framework that can accommodate subjective aesthetic outcomes when they are pre-specified, measured in a standardized manner, and assessed with appropriate safeguards against bias.

## METHODS

### Overview and Registration

This systematic review was conducted following the guidelines set forth by Preferred Reporting Items for Systematic Reviews and Meta-Analyses (PRISMA) and AMSTAR-2.^13,14^ A detailed search strategy was employed across Pubmed and Cochrane to identify relevant literature. Before the review, the protocol was registered on the Prospective Register of Systematic Reviews (PROSPERO ID: CRD42025648513).^15^

### Selection Criteria

The inclusion criteria emphasized RCTs investigating facelift interventions in human subjects, published in academic, peer-reviewed journals. Eligibility was determined solely by study design and topic relevance. The assessment of CONSORT or CONSORT-NPT checklist adherence was conducted retrospectively for the included studies to evaluate the quality of reporting, not as a basis for inclusion or exclusion. The exclusion criteria comprised non-randomized trials, observational research, case reports, and non-interventional study designs involving non-human subjects or those not specifically focused on facelift procedures. Randomized controlled trials were eligible irrespective of publication year; adherence was assessed using CONSORT 2010 (pre-June 20, 2017) or CONSORT-NPT 2017 (on/after June 20, 2017), as appropriate. This methodology guaranteed that only pertinent RCTs focusing on surgical and non-surgical facelift procedures and reporting standards were included.

### Search Strategy

An extensive literature search was conducted to locate studies published from inception to the fifth of February 2025. The searches were conducted across PubMed and Cochrane Library. The Peer Review of Electronic Search Strategies (PRESS) framework informed our search strategy development.^16^ It included terms such as “rhytidoplasty,” “facelift,” “deep plane facelift,” and “Rhytidectomy” using truncations, Medical Subject Headings (MeSH), and Boolean operators to ensure comprehensive retrieval of relevant articles. The reference sections of the included articles were reviewed to identify further relevant publications. English language, human and RCT restrictions were applied.

### Study Selection

Retrieved articles were initially exported to a reference management software EndNote (Clarivate, Philadelphia, PA) (version 20) for deduplication before being imported into Rayyan (Qatar Computing Research Institute, Doha, Qatar) (version 3.0) for screening. Eligibility was determined by 2 reviewers (E.B. and R.P.) who independently screened the titles and abstracts. In the next phase, full-text articles of potentially relevant studies underwent detailed assessment based on the inclusion and exclusion criteria. Any discrepancies were resolved through discussion with a third reviewer (Y.Y.) until a consensus was reached.

### Data Collection and Analysis

The data extraction process was conducted independently by 2 reviewers (Y.Y. and W.J.) using a standardized data extraction form, Microsoft Excel (Microsoft Corp., Redmond, WA) (version 16.54). Data collected included the following variables: study details (author, journal, publication date, and geographical origin), participant demographics, details of facelift interventions, and CONSORT-NPT 2017 adherence for papers published on/after June 20, 2017, and CONSORT 2010 adherence for papers published before that date. All included studies were measured against the criteria outlined in the updated 25-item CONSORT checklist. Full credit for multi-part items requires all sub-parts to be met. A cumulative “CONSORT score” was calculated on a 25-point scale. For each checklist item, compliance was represented by the percentage of studies fulfilling that specific criterion.

Overall CONSORT scores were evaluated against study characteristics, including year of publication, geographical origin, number of authors, and journal indexing status to identify potential correlations. To ensure accuracy, a second reviewer independently verified the scoring. Discrepancies were resolved through discussion or by seeking input from the lead author. Descriptive statistics were used to summarize compliance patterns and identify prevalent reporting deficiencies.

Continuous variables are summarized as mean (SD) or median (IQR). Correlation between CONSORT adherence (%) and journal impact factor and number of authors were tested with Pearson correlation and simple linear regression (adherence as the dependent variable). Two-sided α = 0.05 was prespecified. We report *R*^2^, the correlation coefficient *r*, and *P*-values from the *t*-test of the regression slope = 0 (equivalently, test of *r* = 0). About 95% CIs for *r* were obtained via the Fisher *z* transformation; when distributional assumptions were questionable, we ran a prespecified Spearman rank correlation as a sensitivity check (conclusions unchanged). For descriptive summaries of author count, 95% CIs for the mean were computed using the t distribution (*df* = *n* − 1). Analyses were performed in R v4.3.2 (R Foundation for Statistical Computing, Vienna, Austria).

### Further Critical Appraisal

The Cochrane Risk of Bias 2.0 tool (RoB) was employed to assess the risk of bias in the included studies, and the AMSTAR-2 tool was applied to appraise the methodological quality of systematic reviews.^14,17^ Additionally, results were organized into a table, showcasing the methodological strengths and limitations of each study. Any discrepancies in the quality assessments were resolved via discussion with the lead author (W.J.). The Grading of Recommendations, Assessment, Development, and Evaluation (GRADE) framework was applied to assess the overall quality of evidence.^16^

## RESULTS

**Figure 1.**
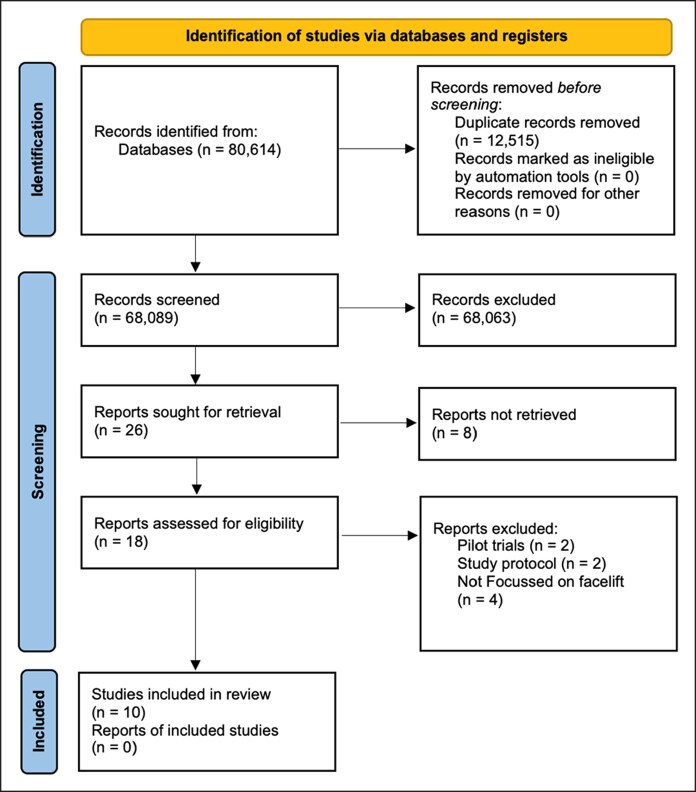
PRISMA flow chart of the selected studies.

The initial database search found 80,614 records, after which 12,515 duplicates were removed, and 68,089 studies' abstracts were screened. The selection process narrowed the pool to 26 papers, which were then subjected to full-text screening. During this stage, 16 studies were excluded because they either were not addressing clinical outcomes related to facelift or lacking clear, descriptive information about the population characteristics or clinical outcomes. The study selection process is summarized in Figure 1 using the PRISMA flow chart.

**Figure 2.**
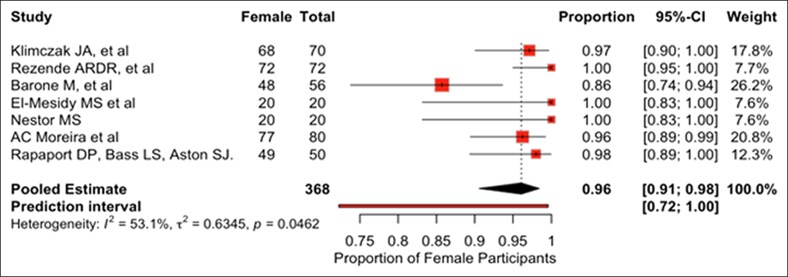
Forest plots of proportion of female patients; squares show study estimates (size ≈ study weight), along with the diamond pooled estimate from a random-effects model.

**Figure 3.**
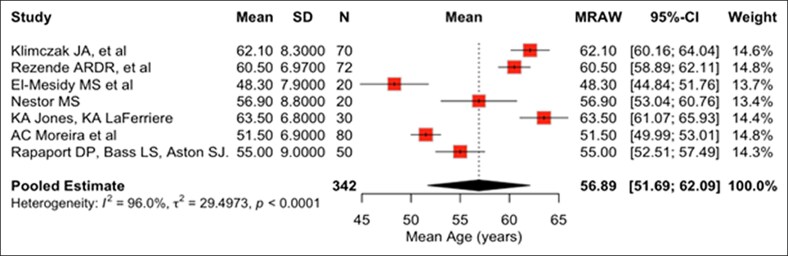
Forest plots of the pooled mean age; squares show study estimates (size ≈ study weight), along with the diamond pooled estimate from a random-effects model.

Table 1 summarizes the characteristics of 10 facelift RCTs published between 1995 and 2025 that were included in this review.^18-27^ USA contributed to 6 studies. The average impact factor of the journals that studies were published in was 2.658 with 2 studies published in journals without an assigned impact factor. Total number of study participants across all studies was 457 with an average of 46 participants per study. The mean age of study participants was 57.6 years. Based on data from 7 studies reporting sex distribution, the pooled proportion of female patients was 96.2%. The pooled mean age, also calculated from 7 studies reporting age, was 56.89 years. Substantial between-study heterogeneity was observed in both pooled analyses (pooled proportion of female patients: *I*² = 53.1%, *τ*² = 0.6345; pooled mean age: *I*² = 96.0%, *τ*² = 29.4973; Q-test *P* < .01 for each). Random-effects models were applied, and pooled estimates should be interpreted with caution given the magnitude of heterogeneity. Forest plots illustrating the proportion of female patients and the pooled mean age across the included studies are shown in Figures 2 and 3, respectively.

**Table 1.** Characteristics of Included Facelift RCTs

Study	Country	Sample (*n*)	Mean Age	M:F	Journal	Author(s) (*n*)	Focus
Klimczak JA^18^	USA	70	62.1 ± 8.3	2:68	Facial Plastic Surgery and Aesthetic Medicine	6	Evaluating the effectiveness of TXA added to tumescent anesthetic in reducing postoperative ecchymosis in rhytidectomy patients
Rezende et al^19^	Brazil	72	60.5 ± 6.97	0:72	*Journal of Plastic, Reconstructive and Aesthetic Surgery*	5	Comparing the efficacy of autologous fibrin glue/PPP vs suction drainage in preventing hematoma and seroma following rhytidectomy procedures
Barone et al^20^	Italy	56	56.5	8:48	*Aesthetic Plastic Surgery*	8	Comparing the outcomes of midface lift alone vs midface lift combined + lipofilling in patients with negative lower eyelid vectors.
El-Mesidy et al^21^	Egypt	20	48.3 ± 7.9	0:20	*Archives of Dermatological Research*	3	Comparing the efficacy of HA cheek fillers and thread lifting in improving the appearance of nasolabial folds.
Nestor^22^	USA	20	56.9 ± 8.8	0:20	*The Journal Of Clinical And Aesthetic Dermatology*	1	Focusing on evaluating the short- and long-term effects of absorbable suspension sutures on facial lift and patient satisfaction.
Jones et al^23^	USA	30	63.5 ± 6.8	Not mentioned	*JAMA Facial Plastic Surgery*	2	Comparing postoperative outcomes in patients undergoing lower rhytidoplasty using two anesthesia methods: combined propofol and ketamine hydrochloride anesthesia with bispectral index monitoring (PKA-BIS protocol) vs inhalational anesthesia (IA), focusing on nausea, vomiting, pain, recovery time, and cost.
Moreira et al^24^	Brazil	80	51.5 ± 6.9	3:77	*The Journal of the Brazilian College of Surgeons*	7	Evaluating the effectiveness of perioperative atenolol in reducing the incidence of hematoma after rhytidoplasty by comparing heart rate, blood pressure, and hematoma formation in patients who received atenolol vs those who did not.
Rapaport et al^25^	USA	50	55 ± 9	1:49	*Plastic and Reconstructive Surgery*	2	Investigating the effectiveness of steroids in reducing postoperative swelling after facialplasty
Seeley et al^26^	USA	29	Not mentioned	Not mentioned	*Archives of Facial Plastic Surgery*	4	Evaluating the efficacy of homeopathic Arnica montana as an antiecchymotic agent in reducing bruising during face-lift surgeries.
Owsley et al^27^	USA	30	56	Not mentioned	*Plastic and Reconstructive Surgery*	3	Investigating whether corticosteroid medication reduces postoperative facial edema in face-lift surgery.

HA, hyaluronic acid; M:F, male:female numbers; n, number; PPP, Platelet-Poor Plasma; TXA, tranexamic acid.

The included RCTs evaluated a clinically heterogeneous range of facelift-related interventions, including traditional rhytidectomy with pharmacologic adjuncts (eg, tranexamic acid, steroids, atenolol), varied anesthesia methods (eg, intravenous vs inhalational), midface lifts with lipofilling, nasolabial fold correction via cheek volume restoration vs thread lifting, absorbable suspension sutures, and autologous fibrin glue/platelet-poor plasma vs suction drainage.

### Percentage Adherence to CONSORT Criteria

**Figure 4.**
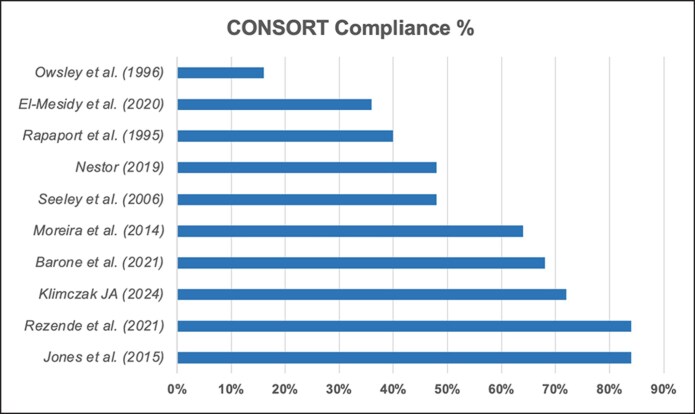
Bar chart showing the percentage adherence to each CONSORT checklist item across the included studies.

Figure 4 illustrates the percentage adherence to each CONSORT checklist item, with a mean compliance of 56%. About 70% of studies identified as RCTs in the title along with 60% of studies providing a structured abstract. About 100% of studies provided detailed descriptions of interventions for each group to allow replication. Similarly, 100% of studies provided detailed descriptions of statistical methods used to compare groups for primary and secondary outcomes. Notably, 90% of the studies disclosed their funding resources. Compliance to external validity and generalizability (50%), mechanism for allocation concealment (50%), how sample size was determined (50%) and method used to generate random allocation sequence (40%) were suboptimal. The lowest reported compliance was with tailored interventions (10%), trial registration (20%), and trial protocol (30%) rising concerns regarding risks of selective reporting bias, and reduced transparency and reproducibility. These findings are summarized in Table 2.

**Table 2.** Per-Item Compliance (%) (Sorted by Percent Compliant, Highest to Lowest)

Checklist Item	Compliance (%)
Detailed intervention description for replication	100
Statistical methods used for primary and secondary outcomes	100
Pre-specified primary and secondary outcomes, how and when assessed	90
Scientific background and explanation of rationale	90
Blinding of participants, care providers, and outcome assessors	90
Study limitations, addressing potential biases	90
Sources of funding and role of funders	90
Description of trial design (parallel, factorial, etc.) and allocation ratio	80
Eligibility criteria for participants and care providers	80
Address issues related to comparator choice, lack of blinding, and expertise differences	80
Identification as a randomized trial in the title	70
Structured summary of trial design, methods, results, and conclusions	60
Recruitment dates and follow-up periods	60
How sample size was determined	50
Mechanism for allocation concealment	50
Flow of participants, losses and exclusions	50
External validity and generalizability	50
Method used to generate random allocation sequence	40
Generalizability according to care providers and centers involved	40
Full trial protocol	30
Trial registration number and registry	20
Details on tailoring interventions to individual participants, standardization, and adherence	10
If blinding was not possible, describe methods to limit bias	N/A
Report the delay between randomization and intervention initiation	N/A

### Relationship Between CONSORT Compliance Score and Journal Impact Factor

**Figure 5.**
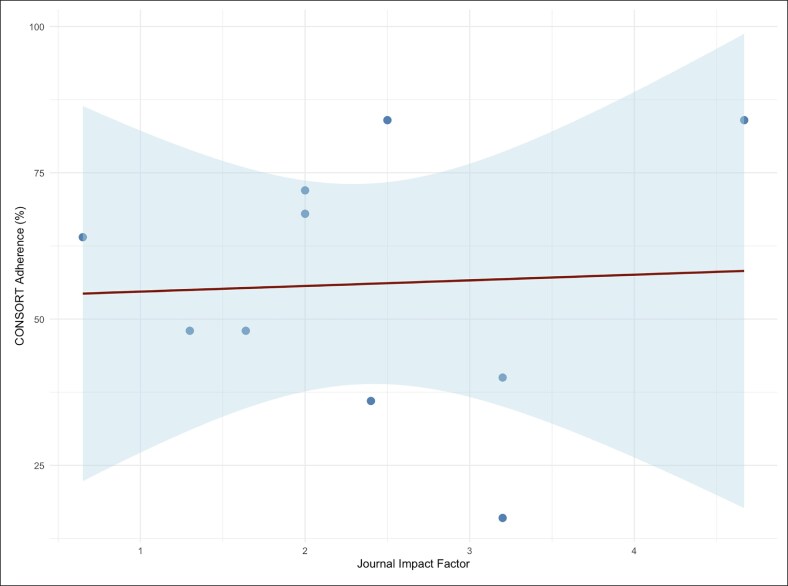
Scatter plot showing the relationship between CONSORT compliance scores and journal impact factors, with a regression line. Spearman correlation was concordant and non-significant.

As shown in Figure 5, analysis of the relationship between CONSORT compliance scores and journal impact factors among the 10 included facelift RCTs revealed a lack of a statistically significant relationship (*P* = .892) and only a very weak positive correlation (*R*² = 0.0024; *r* = 0.05; *P* = .893; 95% CI for *r* −0.60 to 0.66). The mean journal impact factor was 2.36 (range: 1.3-4.67), while the mean CONSORT compliance score was 56% (range: 16%-84%). Notably, the study with the highest CONSORT compliance (84%) was published in a journal with an impact factor of 2.5, while the study in the highest impact journal (impact factor 4.67) also achieved the maximum compliance score (84%). Overall, there was no clear trend linking higher journal impact factors to improved reporting quality, as studies with both high and low compliance scores were distributed across the spectrum of journal impact factors. These findings indicate that, within this sample, publication in higher impact journals does not necessarily correspond to superior adherence to CONSORT guidelines for RCT reporting in facelift research.^7^

### Relationship Between CONSORT Adherence and the Number of Authors

**Figure 6.**
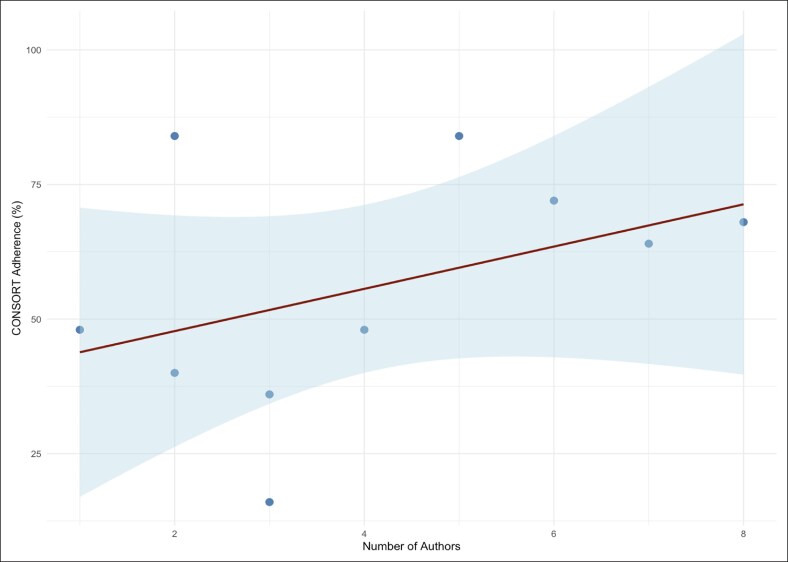
Scatter plot showing the relationship between CONSORT compliance scores and the number of authors, with a regression line. Interpretation: numerically higher adherence with more authors, but not statistically significant; results should be interpreted cautiously given small sample size.

A linear regression analysis was performed to assess the association between the number of authors and CONSORT compliance across facelift RCTs. Although a positive trend was observed, the relationship did not reach statistical significance. Each additional author was associated with a 3.93% point increase in CONSORT adherence (95% CI: −3.13 to 10.98; *P* = .235). The model explained 17.1% of the variance in reporting quality (*R*² = 0.171; *r* = 0.413; *P* = .235; 95% CI for *r* −0.29 to 0.83). A regression plot is presented in Figure 6. These findings suggest that increasing the number of authors may be associated with a modest improvement in reporting quality, but it is not a strong predictor of CONSORT adherence in facelift RCTs.

### Risk of Bias (RoB), GRADE and AMSTAR

The Cochrane RoB assessment found 3 studies to have a low risk of bias, 6 studies raising some concerns and one classified as high risk. The GRADE system was used to assess evidence quality, with 5 studies rated as “Low to Moderate”, 2 studies rated as “Low” and 3 studies rated as “High”. Out of the 5 criteria, Publication Bias was more predominantly compromised, as 70% (*n* = 7) of studies had “Some Concerns”. This can skew the overall evidence base and lead to overestimation of treatment effects. Imprecision domain showed 40% of the studies (*n* = 4) to have “Some Imprecision” and 40% (*n* = 4) to have “Significant Imprecision”. The GRADE framework emphasizes that imprecision can arise from wide confidence intervals and small sample sizes, which may lead to uncertainty in the effect estimates. A total of 70% (*n* = 7) of the imprecise studies had small sample sizes which can result in insufficient data to provide precise estimates of the effect, thereby increasing uncertainty.^28^ However, no studies were downgraded for Indirectness or Inconsistency, supporting the generalizability of the findings. Supplemental Table 1 better summarizes our GRADE assessment.

Concerning AMSTAR-2 evaluation of our own systematic review, we prospectively registered a protocol, performed independent duplicate screening and data extraction, conducted a structured, database-driven search informed by Peer Review of Electronic Search Strategies (PRESS), and assessed risk of bias for all included RCTs and considered RoB in our interpretation. Domains related to meta-analysis and small-study effects were not applicable because no quantitative synthesis was undertaken. A detailed summary of the AMSTAR-2 assessment results is provided in Supplemental Table 2.

## DISCUSSION

Despite the global popularity and routine performance of facelift surgery, this review highlights a significant gap in high-level evidence supporting its efficacy and safety. Only 10 RCTs were identified in the published literature, a surprisingly low number for a procedure that is both widely practised and technically diverse.

Several factors may contribute to this evidence gap. First, ethical and practical challenges make RCTs difficult to conduct in aesthetic surgery. As facelifts are elective and patient-driven, randomizing individuals to different surgical techniques, particularly those perceived as superior or inferior, can raise ethical concerns and hinder patient enrolment. Surgeons may also be reluctant to deviate from their preferred techniques for the sake of trial standardization. Second, the heterogeneity of facelift techniques and outcomes presents methodological barriers. Variations in surgical approach, surgeon expertise, and patient anatomy make it difficult to design studies with consistent interventions. Furthermore, aesthetic outcomes are inherently subjective and lack universally accepted measurement tools, complicating the establishment of standardized endpoints necessary for high-quality trials. Together, these challenges explain the deviation to alternative study designs, such as well-conducted prospective cohort studies, expert consensus guidelines, or the development of validated aesthetic outcome measures, to strengthen the evidence base in facelift surgery. This disparity highlights the persistent gap between surgical practice and evidence-based standards in aesthetic surgery, where innovations are frequently adopted without rigorous scientific validation or standardized reporting, as noted by Wood et al^29^ in their appraisal of evidence generation in plastic surgery research.

While the number of studies per country was limited, preliminary observations suggested some variability in adherence rates. These differences may reflect disparities in institutional research culture, regulatory expectations, or journal editorial standards across regions. Further investigation into national trends could reveal whether certain countries consistently produce higher-quality surgical trials, potentially setting a benchmark for global reporting practices in aesthetic surgery.^30^

Building upon these observations, our analysis shifts focus from the number of existing facelift RCTs to their reporting quality. Using the CONSORT checklist as a reference, we evaluated each study's methodological transparency to better understand the current evidence landscape in aesthetic facial surgery.

Our systematic review revealed a mean CONSORT adherence score of 56%. While domains such as intervention descriptions and statistical analyses were consistently well reported, key elements, including trial registration (20%) and protocol availability (30%), remained underrepresented. These gaps raise concerns about transparency, selective reporting, and reproducibility, even in a high-visibility and innovation-driven field such as aesthetic facial surgery.^31,32^

When pooling the proportion of female patients and the pooled mean age, heterogeneity was substantial. This likely reflects clinical and methodological diversity, differences in techniques/adjuncts, surgeon experience and learning curves, case mix and baseline anatomy, outcome definitions/timepoints, and limited blinding/measurement tools. With few trials, subgroup analysis or meta-regression was not feasible. We therefore interpret pooled estimates cautiously and recommend standardizing endpoints, capture protocols, and timepoints in future studies to reduce between-study variance.

Our quality appraisal using the Cochrane RoB tool and GRADE framework further highlighted the fragility of the existing evidence. Only 3 of the 10 studies were rated as having low risk of bias, while 7 showed either high risk or some concerns; particularly in the domains of publication bias and imprecision. Notably, small sample sizes were a common contributor to lower GRADE ratings, reinforcing concerns about the robustness and reproducibility of available facelift RCTs.

Given that our review is, to the best of our knowledge, the first to address this specific question, no other systematic reviews were available for comparative analysis or inclusion. AMSTAR-2 domains were used to inform our methodological transparency and adherence to best practices, not as a formal self-assessment tool.

The observed variability in reporting is consistent with findings in other surgical subspecialties, including ophthalmology and general plastic surgery. Yao et al (2014) reported similarly poor compliance in ophthalmic surgical trials, with an average score of 8.9/23 (∼39%), emphasizing that inadequate reporting is not unique to the aesthetic domain. Moreover, prior reviews in plastic surgery have highlighted systemic gaps in adherence to CONSORT, particularly in non-pharmacological interventions, despite the availability of tailored guidelines since 2008.^8,32^

Interestingly, this review identified only weak correlations between CONSORT adherence and both journal impact factor (*R*² = 0.0024) and number of authors (*R*² = 0.171). These findings suggest that neither prestige nor author counts are reliable predictor of methodological transparency. This echoes similar conclusions from studies in orthopedic and cardiovascular surgery, where journal metrics and team size failed to significantly influence reporting quality.^33,34^ While the CONSORT statement remains the recognized standard for reporting randomized trials, prior studies have found that even top-tier journals such as New England Journal of Medicine (NEJM), Journal of the American Medical Association (JAMA) and The Lancet report variable adherence rates—ranging from 66% to 89%—with gaps in blinding, randomization, and outcome transparency still observed.^35^ Collectively, these indicate that internal study design practices, rather than external prestige, may be more determinative of reporting quality.

The persistent underreporting of key trial components has direct consequences for clinical translation. Without clear documentation of randomization, allocation concealment, and sample size justification, the internal validity of RCTs is compromised. In facelift surgery, where procedural nuances and patient expectations are uniquely high, methodological rigor becomes crucial not only for evidence synthesis but also for informing ethical patient counseling and resource allocation.^31,36^

To translate these insights into practice, we propose a practical roadmap for future facelift RCTs. First, mandatory pre-registration on platforms like ClinicalTrials.gov should be enforced by journals and funding bodies to mitigate selective reporting bias and enhance transparency. Second, trials should prioritize validated aesthetic outcome tools, such as the FACE-Q scales or standardized photographic assessments with inter-rater reliability metrics, to ensure subjective endpoints like patient satisfaction and facial rejuvenation are measured meaningfully. Third, multicenter collaborations, potentially facilitated through professional societies, can address sample size limitations by pooling diverse patient cohorts, improving generalizability, and distributing expertise across institutions.

Improving methodological transparency requires journals to go beyond passive endorsement of reporting guidelines. In aesthetic surgery, where robust RCTs remain limited, embedding the CONSORT checklist into editorial workflows could serve as a critical safeguard for study quality. Additionally, researchers must be trained in trial reporting standards, especially in surgical disciplines where technical complexity may obscure methodological transparency. Institutional mandates and funding bodies should also condition support on pre-registration and protocol publication.^37-39^

The included RCTs encompassed a diverse range of facelift-related interventions, from surgical techniques like traditional rhytidectomy with adjuncts and varied anesthesia methods, to minimally invasive approaches such as thread lifts, fibrin glue, Arnica montana, atenolol, and corticosteroids. This clinical heterogeneity highlights the challenge of standardizing outcomes and reinforces the importance of transparent reporting to help surgeons interpret and apply the evidence in daily practice.

This review has several limitations. First, the relatively small number of eligible facelifts RCTs (*n* = 10) may limit the generalizability of our findings, although it reflects the overall scarcity of high-level evidence in this field. The paper focused on facelift RCTs reported in English, potentially excluding relevant work from non-English-speaking countries. This language restriction may introduce language publication bias by potentially excluding relevant work from non-English-speaking countries, which could underrepresent diverse surgical techniques, patient populations, or outcomes. We also did not assess unpublished or gray literature, which may contain trials that were registered but not reported due to negative findings. While our analysis provides valuable insights into current reporting practices, it is descriptive in nature and not intended to support definitive clinical guidance. The small sample size, combined with considerable heterogeneity in study interventions and outcomes, further limits the ability to perform meta-analyses or draw robust comparative conclusions. Second, the evaluation of CONSORT compliance was inherently subjective, despite the use of independent reviewers and consensus discussions. The variability in CONSORT adherence may reflect journal policy or author awareness rather than inherent trial quality. Third, our analysis did not assess the clinical outcomes or effect sizes reported in the trials, focusing instead solely on methodological reporting. Another notable limitation observed across the included studies is the predominance of short-term, complication-focused outcome measures; such as edema, bruising, and hematoma, rather than outcomes aligned with the primary goals of facelift surgery, namely aesthetic improvement and patient satisfaction. Only a minority of trials evaluated these patient-centred or appearance-related endpoints. This gap in outcome selection limits the clinical applicability of the evidence, as complication rates alone do not reflect the true success of aesthetic procedures. However, it is essential to distinguish between subjective and objective assessments when quantifying results. Patient-reported outcomes, such as those from validated questionnaires, measure satisfaction and perceived improvements but remain inherently subjective and do not provide a quantitative evaluation of physical changes, such as how closely the result approximates an “ideal normal” anatomy. Although patient satisfaction is crucial for patient-centered care, relying solely on these without complementary objective metrics (eg, standardized imaging or anthropometric measurements) may limit the ability to objectively assess surgical efficacy. Future facelift RCTs should incorporate standardized, validated aesthetic outcome measures and patient-reported outcome instruments to better capture the multidimensional impact of surgery. This aligns with broader calls within the surgical literature for more meaningful and reproducible endpoints in aesthetic research.^29,31^ Additionally, while studies were excluded if they lacked fundamental information such as population characteristics or outcome data, this was done solely to ensure that CONSORT adherence could be objectively and consistently assessed. We acknowledge that this may introduce a degree of selection bias toward more completely reported studies, and we have highlighted this as a potential limitation in interpreting overall compliance rates. Moreover, most trials prioritized short-term complications rather than the outcomes that matter most, the overall aesthetic improvement and patient satisfaction. While inherently subjective, these endpoints can be assessed rigorously by pre-specifying outcomes and timepoints; standardizing photography and rating rubrics; using blinded, calibrated multi-rater panels with reported inter-rater reliability; incorporating validated patient-reported measures with MCIDs; and powering studies accordingly. Embedding these practices within CONSORT enhances transparency while preserving the central role of expert judgment and patient perception. Lastly, while the inclusion of studies published before 2017 allowed us to analyze reporting trends over time, it introduced variability due to evolving guideline standards. While this review focuses on RCTs and CONSORT adherence, it is important to recognise that randomised trials account for fewer than 5% of published studies in plastic surgery.^40^ The majority of studies in the field are observational and thus subject to the Strengthening the Reporting of Observational Studies in Epidemiology (STROBE) reporting guidelines. Agha et al (2016) found that reporting quality in observational plastic surgery studies was similarly suboptimal, with poor adherence to key STROBE items.^41^ This parallel highlights a broader issue of inadequate methodological transparency across study designs in plastic surgery and reinforces the need for more rigorous enforcement of reporting standards by journals, editors, and peer reviewers alike. It is also important to note that an updated version (CONSORT 2025 extension) was released during our project. While our assessment reflects adherence to the 2017 framework, future research should consider aligning with the latest 2025 recommendations to ensure up-to-date reporting standards and methodological rigor.^42^

Ethical constraints and technical heterogeneity will always complicate surgical RCTs; yet, these barriers do not excuse inadequate documentation of the methods that are used. Robust prospective registries, standardized aesthetic outcome measures, and well-powered cohort studies can complement RCTs, but only if they, too, meet high reporting standards. To narrow the gap between clinical practice and evidence-based care, stakeholders must act in concert. Investigators should pre-register trials, publish protocols, and adopt CONSORT checklists as routine practice. Journals and peer reviewers should enforce adherence as a condition of publication, and funding bodies should require compliance as part of grant oversight. By embracing these measures, the facelift community can move beyond tradition-driven innovation toward a transparent, reproducible, and patient-centred body of clinical research that truly informs surgical decision-making.

## CONCLUSIONS

This systematic review highlights an important opportunity in contemporary aesthetic surgery: while demand for facelift procedures continues to rise and progress has been made through the conduct of a small but growing number of RCTs, the supporting evidence base remains limited and inconsistently reported, underscoring the need for continued methodological rigor. Even within this small cohort (10 RCTs), mean adherence to the CONSORT checklist was just 56%, and particularly low compliance in areas such as tailored interventions (10%), trial registration (20%), and trial protocol (30%). These omissions compromise transparency, restrict reproducibility, and hinder reliable evidence synthesis.

## Supplemental Material

This article contains supplemental material located online at https://doi.org/10.1093/asjof/ojaf153.

## Supplementary Material

ojaf153_Supplementary_Data
